# The translational potential of miR-26 in atherosclerosis and development of agents for its target genes ACC1/2, COL1A1, CPT1A, FBP1, DGAT2, and SMAD7

**DOI:** 10.1186/s12933-024-02119-z

**Published:** 2024-01-09

**Authors:** Wujun Chen, Xiaolin Wu, Jianxia Hu, Xiaolei Liu, Zhu Guo, Jianfeng Wu, Yingchun Shao, Minglu Hao, Shuangshuang Zhang, Weichao Hu, Yanhong Wang, Miao Zhang, Meng Zhu, Chao Wang, Yudong Wu, Jie Wang, Dongming Xing

**Affiliations:** 1 Cancer Institute, Department of Orthopaedics, The Affiliated Hospital of Qingdao University, Qingdao University, Qingdao Cancer Institute, Qingdao, 266071 Shandong China; 2 Department of Endocrinology, The Affiliated Hospital of Qingdao University, Qingdao, 266000 Shandong China; 3 Department of Gastrointestinal Surgery, The Affiliated Hospital of Qingdao University, Qingdao, 266000 Shandong China; 4 Department of Cardiology, The Second Affiliated Hospital, Hengyang Medical School, University of South China, Key Laboratory of Heart Failure Prevention & Treatment of Hengyang, Clinical Medicine Research Center of Arteriosclerotic Disease of Hunan Province, Hengyang, 421001 Hunan China; 5 Department of Endocrinology, Qilu Hospital (Qingdao), Cheeloo College of Medicine, Shandong University, Qingdao, 266000 Shandong China; 6 Department of Neurosurgery, The Affiliated Hospital of Qingdao University, Qingdao, 266071 Shandong China; 7 School of Life Sciences, Tsinghua University, Beijing, 100084 China

**Keywords:** miR-26, IFN-α therapy, COL1A1, FBP1, DGAT2, SMAD7

## Abstract

Atherosclerosis is one of the leading causes of death worldwide. miR-26 is a potential biomarker of atherosclerosis. Standardized diagnostic tests for miR-26 (MIR26-DX) have been developed, but the fastest progress has been in predicting the efficacy of IFN-α therapy for hepatocellular carcinoma (HCC, phase 3). MiR-26 slows atherosclerosis development by suppressing ACC1/2, ACLY, ACSL3/4, ALDH3A2, ALPL, BMP2, CD36, COL1A1, CPT1A, CTGF, DGAT2, EHHADH, FAS, FBP1, GATA4, GSK3β, G6PC, Gys2, HMGA1, HMGB1, LDLR, LIPC, IL-1β, IL-6, JAG2, KCNJ2, MALT1, β-MHC, NF-κB, PCK1, PLCβ1, PYGL, RUNX2, SCD1, SMAD1/4/5/7, SREBF1, TAB3, TAK1, TCF7L2, and TNF-α expression. Many agents targeting these genes, such as the ACC1/2 inhibitors GS-0976, PF-05221304, and MK-4074; the DGAT2 inhibitors IONIS-DGAT2Rx, PF-06427878, PF-0685571, and PF-07202954; the COL1A1 inhibitor HT-100; the stimulants ^68^Ga-CBP8 and RCT-01; the CPT1A inhibitors etomoxir, perhexiline, and teglicar; the FBP1 inhibitors CS-917 and MB07803; and the SMAD7 inhibitor mongersen, have been investigated in clinical trials. Interestingly, miR-26 better reduced intima-media thickness (IMT) than PCSK9 or CT-1 knockout. Many PCSK9 inhibitors, including alirocumab, evolocumab, inclisiran, AZD8233, Civi-007, MK-0616, and LIB003, have been investigated in clinical trials. Recombinant CT-1 was also investigated in clinical trials. Therefore, miR-26 is a promising target for agent development. miR-26 promotes foam cell formation by reducing ABCA1 and ARL4C expression. Multiple materials can be used to deliver miR-26, but it is unclear which material is most suitable for mass production and clinical applications. This review focuses on the potential use of miR-26 in treating atherosclerosis to support the development of agents targeting it.

## Introduction

Cardiovascular disease (CVD) is one of the leading causes of death worldwide [1]. The formation of atherosclerotic plaques obstructs blood flow to organs, the clinical manifestations of which include myocardial infarction (MI), ischemic heart disease (IHD), and stroke, which are the main causes of CVD [2–5]. Atherosclerosis is a chronic inflammatory disease caused by the dysregulation of lipid metabolism. When the blood low-density lipoprotein (LDL)-c concentration is higher than the physiological level, LDL-c passively diffuses from the vascular lumen to the intima and is oxidized to form ox-LDL in response to reactive oxygen species (ROS). Macrophages engulf a large amount of ox-LDL, and their efflux is blocked, resulting in the accumulation of cholesterol in macrophages and the formation of foam cells [6, 7]. Foam cells recruit vascular smooth muscle cells (VSMCs) from the middle membrane. Migrating VSMCs form fibrous caps on plaques by secreting matrix metalloproteinase 2 (MMP2), MMP9, collagen, and elastin, leading to persistent inflammation and endothelial dysfunction. Over time, the plaque gradually becomes large enough to damage the arterial cavity, blocking blood flow to the tissue and causing tissue ischemia or plaque rupture to form blood clots. Therefore, timely inhibition of lipid metabolism disorders, the inflammatory response, and VSMC migration is the focus of atherosclerosis prevention and treatment of [8–12].

MiR-26, a vertebrate-specific miRNA, includes miR-26a (also named miR-26a-5p) and miR-26b (also named miR-26b-5p). MiR-26a/b is expressed in different tissues, including the spleen, brain, kidney, liver, lung, heart, testis, adipose tissue, and macrophages [13]. The mature sequences of miR-26a and miR-26b are identical except for two base differences. MiR-26a includes miR-26a-1 and miR-26a-2. miR-26a-1 is located in the intron of the C-terminal domain of RNA polymerase II polypeptide A small phosphatase-like (CTDSPL; also known as C3orf8, HYA22, PSR1, RBSP3, and SCP3). MiR-26a-2 is located in the intron of the C-terminal domain of RNA polymerase II polypeptide A small phosphatase 2 (CTDSP2; also known as OS4, PSR2, and SCP2). MiR-26b is located in the intron of CTDSP1 (also known as NIF3, NLI-IF, NLIIF, and SCP1). The mature sequences of miR-26a-1 and miR-26a-2 are identical [14]. MiR-26 decreases atherosclerosis development by regulating many genes, including ATP-binding cassette transporter A1 (ABCA1), acetyl-CoA-carboxylase 1 (ACC1), ACC2, ATP-citrate lyase (ACLY), Acyl-CoA synthetase long-chain 3 (ACSL3), ACSL4, aldehyde dehydrogenase 3 family member A2 (ALDH3A2, also named fatty aldehyde dehydrogenase (FALDH)), alkaline phosphatase (ALPL), ARF-like 7 (ARL7, also named ADP-ribosylation factor-like 4C (ARL4C)), β-myosin heavy chain (β-MHC, also named MYH7), bone morphogenetic protein 2 (BMP2), cluster of differentiation 36 (CD36), collagen I (COL1A1), carnitine palmitoyl-transferase 1A (CPT1A), connective tissue growth factor (CTGF), diacylglycerol acyltransferase 2 (DGAT2), enoyl-CoA hydratase/3-hydroxyacyl-CoA dehydrogenase (EHHADH), fatty acid synthase (FASN, also named FAS), fructose-1,6-bisphosphatase (FBP1, also named FBPase), GATA binding protein 4 (GATA4), glucose-6-phosphatase (G6PC, also named G6PC1, G6Pase, and G6Pase-α), glycogen synthase kinase-3β (GSK-3β), glycogen synthase 2 (Gys2), high mobility group A1 (HMGA1), HMGB1, interleukin (IL)-1 beta (IL-1β), IL-6, jagged canonical Notch ligand 2 (JAG2), potassium inwardly rectifying channel subfamily J member 2 (KCNJ_2_), low-density lipoprotein receptor (LDLR), lipase C hepatic type (LIPC), mucosa-associated lymphoid tissue lymphoma translocation protein 1 (MALT1), nuclear factor κB (NF-κB), carboxykinase (PCK1), phospholipase C beta (PLCβ1), glycogen phosphorylase L (PYGL), runt-related protein 2 (RUNX2), stearoyl-coenzyme A desaturase 1 (SCD1), mothers against decapentaplegic homolog 1 (SMAD1), SMAD4, SMAD5, SMAD7, sterol regulatory element-binding transcription factor 1 (SREBF1), TGFβ-activated kinase binding protein 3 (TAB3), transforming growth factor β (TGFβ)-activated kinase-1 (TAK1, also named MAP3K7), transcription factor 7 like 2 (TCF7L2, also named TCF-4), and tumor necrosis factor alpha (TNF-α) [15–25]. Many drugs have been brought to market or developed in clinical trials by targeting these genes, suggesting that targeting these genes can greatly improve the success rate of drug development [22, 26–29]. The value of miR-26 has also been investigated in clinical trials. This review focuses on the role and mechanism of action of miR-26 in atherosclerosis, including the detection value of miR-26 and the potential development of drugs targeting its downstream genes, such as ACC1/2, COL1A1, CPT1A, FBP1, DGAT2, and SMAD7**,** to provide insight for drug development.

## The clinical value of miR-26 quantification

### Atherosclerosis

The diagnostic value of miR-26 in atherosclerosis has been investigated in clinical trials and preclinical models [30–35]. The serum miR-26 level was negatively associated with serum total cholesterol (TC), triglycerides (TG), and LDL-C and positively associated with HDL-C in patients with carotid atherosclerosis (CAS) and in apoE−/− mice [36]. The miR-26 level in exosomes produced by adipose-derived stem cells (ADSC-exos) was negatively associated with TNF-α, IL-6, and IL-1β, suggesting that the ADSC-exo miR-26 level is a potential biomarker for the diagnosis of lipids and inflammatory factors (Table 1) [36]. Clinical studies on the diagnostic value of miR-26 in atherosclerosis are limited. Biomarkers should be sensitive and specific for diagnosing the disease state. More studies are needed to confirm the clinical translatability of miR-26.

**Table 1:** The diagnostic value of miR-26 in diseases in clinical trials

Diseases	Status	Refs.
Atherosclerosis	Completed	[36]
HCC	Phase 3	[39]
T1DM	Completed	[50]
T2DM	Completed	[50]
MINOCA	Recruiting	[51]
Leukoencephalopathy	Recruiting	[52]
EHOA and PsA	Completed	[53]
Prostate cancer	Recruiting	[54]
Atopy in children over time	Active, not recruiting	[55]

The information was obtained from ClinicalTrials and Pubmed

*HCC* hepatocellular carcinoma, *EHOA* erosive hand osteoarthritis, *MINOCA* myocardial infarction with nonobstructive coronary arteries, *PsA* psoriatic arthritis, *T1DM* type 1 diabetes mellitus

LIPC, a miR-26 target gene, encodes hepatic lipase (HL) and promotes the hydrolysis of TG. LIPC not only remodels LDL and HDL but also enhances lipid and lipoprotein uptake [37]. Interestingly, LIPC gene mutation is the second most common cause of familial hypocholesterolemia, after only angiopoietin-like 3 (ANGPTL3) [38], suggesting that LIPC is a promising target for the diagnosis of familial hypocholesterolemia.

### Prediction of the efficacy of IFN-α

**Fig. 1:**
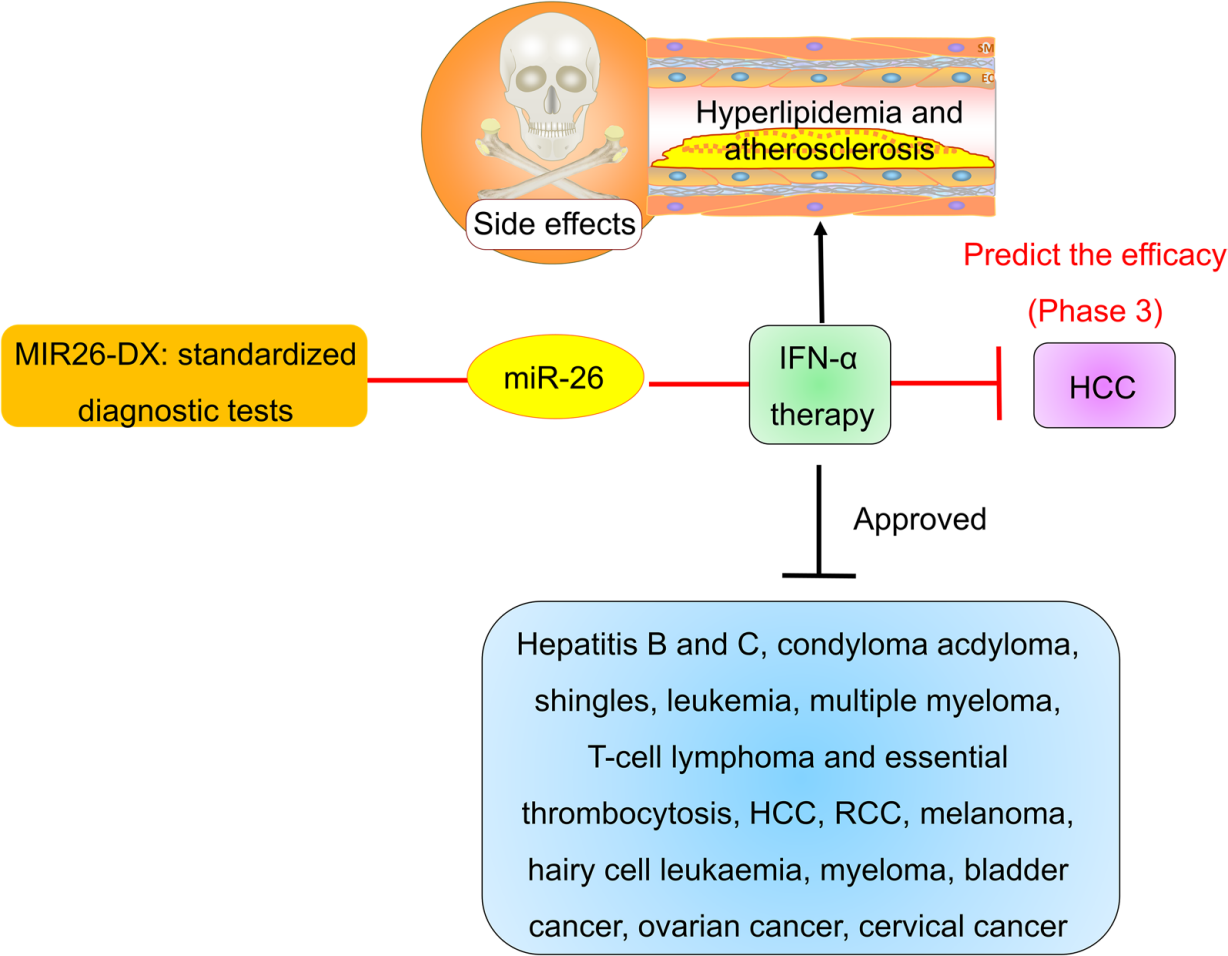
The clinical detection value of miR-26 in HCC. IFN-α therapy was more effective in HCC patients with low miR-26 expression. HCC, hepatocellular carcinoma; IFN-α, interferon-α; MIR26-DX, standardized diagnostic tests for miR-26; RCC, renal cell cancer

The effectiveness of interferon-α (IFN-α) in patients with low miR-26 expression in tumors after curative resection of hepatocellular carcinoma (HCC) is under phase 3 clinical investigation. IFN-α therapy is expected to improve disease-free survival in patients with low miR-26 expression by inhibiting tumor recurrence [39]. Compared with patients with HCC with high expression of miR-26, patients with low-miR-26-expressing tumors had shorter overall survival but a better response to IFN-α therapy (n = 489) [40–42], suggesting that IFN-α therapy is more effective in HCC patients with low miR-26 expression. The use of miR-26 as a predictive marker of therapeutic responsiveness has also received patent protection (WO2009152300A1). Standardized diagnostic tests for miR-26 (MIR26-DX) have also been developed [42]. IFN-α therapy has been used in more than 40 countries to treat viral infections, such as hepatitis B and C; condyloma; shingles; hematological diseases such as leukemia, multiple myeloma, T-cell lymphoma and essential thrombocytosis; and cancers such as renal cell cancer (RCC), melanoma, HCC, hairy cell leukemia, myeloma, bladder cancer, ovarian cancer, and cervical cancer (Fig. 1) [43–45]. The downside is that IFN-α therapy can promote hyperlipidemia and atherosclerosis [46]. The cardiovascular side effects of IFN-α therapy should be noted if it is to be applied clinically [47]. Therefore, miR-26 may be the first miRNA whose expression is considered in the decision to use IFN-α treatment for HCC.

In fact, IFN-α suppressed HCC proliferation and invasion by inducing miR-26a and suppressing the expression of its target enhancer of zeste homolog 2 (EZH2) [48]. miR-26a also increased IFN-α expression by targeting ubiquitin-specific protease 3 (USP3), which is a negative regulator of IFN-α [49], suggesting that IFN-α and miR-26a inhibit the development of HCC by forming positive feedback loops. However, IFN-α also promoted miR-26a degradation by increasing polyribonucleotide nucleotidyltransferase 1 (PNPT1) and its thus the DNA demethylation that it mediated. More studies are needed to confirm the role of IFN-α in regulating miR-26a expression.

### T2DM, T1DM, MINOCA, leukoencephalopathy, EHOA, PsA, prostate cancer, and atopy

The plasma-derived exosomal miR-26a level was reduced in patients with type 2 diabetes mellitus (T2DM). miR-26a also promoted insulin sensitivity. Therefore, exosomal miR-26a is not only a potential biomarker for T2DM but also a potential therapeutic target. However, serum miR-26a was increased in children with T1DM [50]. The diagnostic value of miR-26 in diseases, including myocardial infarction with nonobstructive coronary arteries (MINOCA) [51], leukoencephalopathy [52], erosive hand osteoarthritis (EHOA) and psoriatic arthritis (PsA) [53], prostate cancer [54], and atopy in children over time (including atopy, atopic dermatitis eczema, wheezing and food allergy in infants), is under clinical research (Table 1) [55], the results of which have not been disclosed.

## The role and mechanism of action of miR-26 in atherosclerosis

**Fig. 2:**
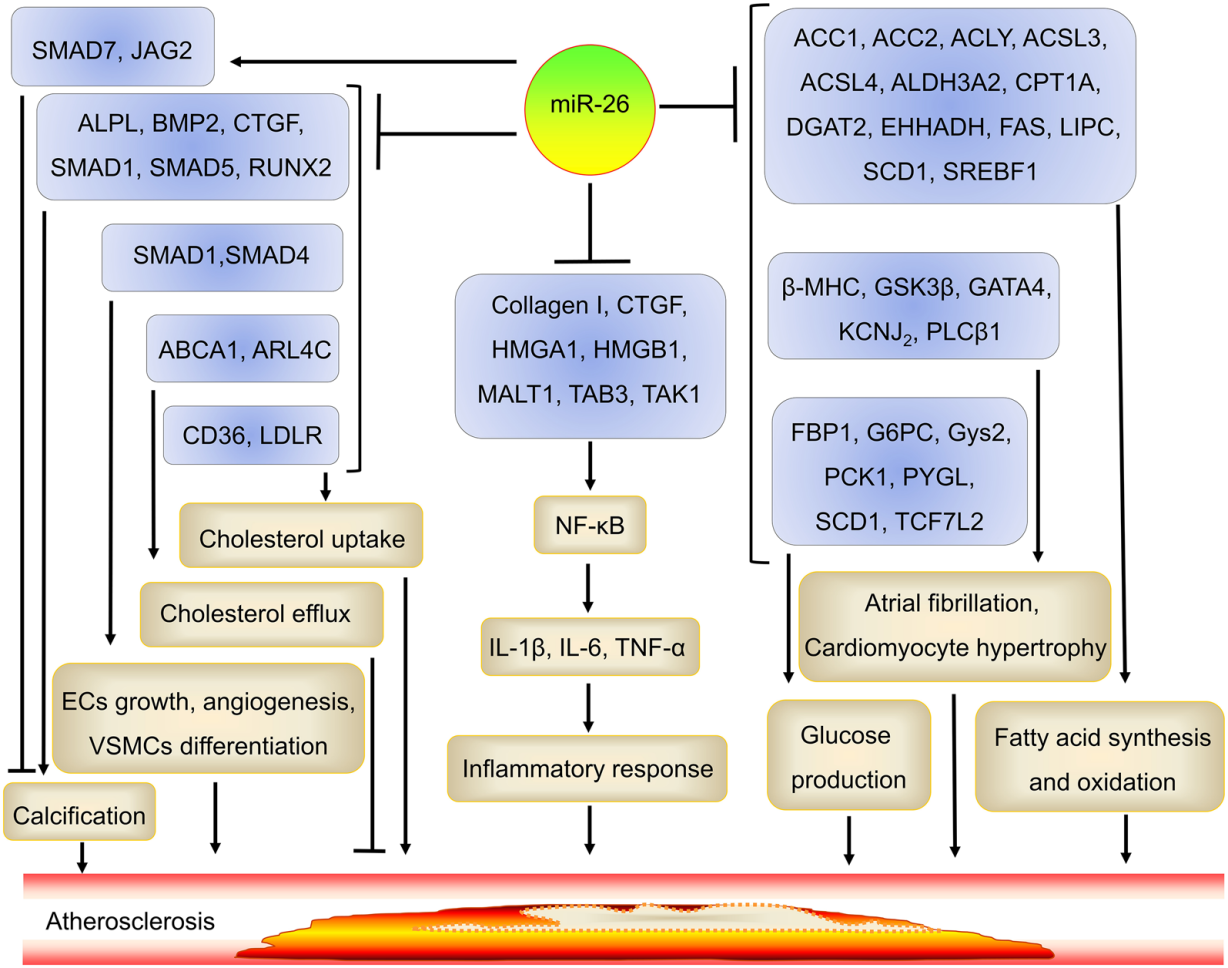
The role and mechanism of miR-26 in atherosclerosis. MiR-26 regulates the development of atherosclerosis through multiple mechanisms, including suppressing vascular calcification, EC growth, angiogenesis, and VSMC differentiation, cholesterol uptake, inflammatory response, glucose production, and FA synthesis and oxidation. *ABCA1* ATP-binding cassette transporter A1, *ACC1* acetyl-CoA-carboxylase 1, *ACLY* ATP-citrate lyase, *ACSL3* Acyl-CoA synthetase long-chain 3, *ALDH3A2* aldehyde dehydrogenase 3 family member A2, *ALPL* alkaline phosphatase, *ARL4C* ARF-like 7, *β-MHC* β-myosin heavy chain, *BMP2* bone morphogenetic protein 2, *CD36* cluster of differentiation 36, *CPT1A* carnitine palmitoyl-transferase 1A, *CTGF* connective tissue growth factor, *DGAT2* diacylglycerol acyltransferase 2, *EHHADH* enoyl-CoA hydratase/3-hydroxyacyl-CoA dehydrogenase, *FAS* fatty acid synthase, *FBP1* fructose-1,6-bisphosphatase, *GATA4* GATA binding protein 4, *G6PC* glucose-6-phosphatase, *GSK3β* glycogen synthase kinase-3β, *Gys2* glycogen synthase 2, *HMGA1* high mobility group A1, *IL-1β* interleukin (IL)-1 beta, *JAG2* jagged canonical Notch ligand 2, *KCNJ*_*2*_ potassium inwardly rectifying channel subfamily J member 2, *LDLR* low-density lipoprotein receptor, *LIPC* lipase C hepatic type, *MALT1* mucosa-associated lymphoid tissue lymphoma translocation protein 1, *NF-κB* nuclear factor κB, *PCK1* carboxykinase, *PLCβ1* phospholipase C beta, *PYGL* glycogen phosphorylase L, *RUNX2* runt-related protein 2, *SCD1* stearoyl-coenzyme A desaturase 1, *SMAD1* mothers against decapentaplegic homolog 1, *SREBF1* sterol regulatory element-binding transcription factor 1, *TAB3* TGFβ-activated kinase binding protein 3, *TCF7L2* transcription factor 7 like 2, *TNF-α* tumor necrosis factor alpha

MiR-26 has become a novel target for the treatment of atherosclerosis. Overexpression of miR-26 in ADSC-Exos decreased the size of atherosclerotic plaques by correcting the changes in the serum lipid and proinflammatory factor levels in the apoE−/− mice. Mechanistically, miR-26 decreases IL-1β, IL-6, and TNF-α levels by suppressing NF-κB activity through the targeting of COL1A1, CTGF, HMGA1, HMGB1, MALT1, TAB3, and TAK1 [15–17, 26, 27]. MiR-26 suppresses endothelial cell (EC) growth, angiogenesis, and VSMC differentiation by targeting SMAD1 and SMAD4 [18, 28, 56, 57]. MiR-26 can also suppress atrial fibrillation (AF) and cardiomyocyte hypertrophy by targeting β-MHC, GSK3β, GATA4, KCNJ_2_, and PLCβ1 [58]. MiR-26a suppresses vascular and aortic valve calcification by suppressing the expression of pro-calcification genes, including ALPL, BMP2, CTGF, SMAD1, SMAD5, and RUNX2, and increasing the expression of anti-calcification genes, including SMAD7 and JAG2 [19–21]. MiR-26a has decreased hepatic glucose production by suppressing the expression of gluconeogenesis- and glycogen metabolism-related genes, such as FBP1, G6PC, Gys2, PCK1, PYGL, SCD1, and TCF7L2 [22] and suppressed fatty acid (FA) synthesis and oxidation by suppressing the expression of ACC1, ACC2, ACLY, ACSL3, ACSL4, ALDH3A2, CPT1A, DGAT2, EHHADH, FAS, LIPC, SCD1, and SREBF1. MiR-26a can suppress cholesterol uptake by suppressing CD36 and LDLR expression [22]. Therefore, miR-26 decreases atherosclerotic plaque size by regulating lipid metabolism, the inflammatory response, angiogenesis, cell differentiation, calcification, glucose metabolism, and insulin signaling-related genes (Fig. 2). However, miR-26 also suppresses cholesterol efflux to promote foam cell formation by binding and suppressing ABCA1 and ARL4C in vitro, as observed in RAW264.7 cells, THP-1 cells, and HepG2 cells [23, 59–61], suggesting that the formation of foam cells induced by miR-26 may weaken its ability to reduce atherosclerosis. Notably, the role of miR-26 in foam cell formation has not been investigated in vivo.

## The clinical application of miR-26 target genes, with a focus on ACC1/2, COL1A1, CPT1A, DGAT2, FBP1, and SMAD7

### ACC1, ACC2, and DGAT2

ACC1/2 catalyzes the conversion of acetyl-CoA to malonyl-CoA, which is the raw material for TG biosynthesis [62, 63]. Many inhibitors, such as GS-0976 (also named firsocostat, NDI-010976) [64, 65], PF-05221304 (also named clesacostat) [66, 67], MK-4074 [68], ND-654 [69], and ND-646 [70, 71], have been investigated in preclinical and clinical trials (Table 2), suggesting that ACC1/2 is a promising target for drug development. However, hypertriglyceridemia is a common adverse effect of ACC1/2 inhibitors that limits their clinical development [72].

**Table 2:** The drugs were approved or investigated in clinical trials by targeting ACC1/2, COL1A1, CPT1A, CT-1, FBP1, PCSK9, and SMAD7

Drug names	Structure/introduce	Target	Diseases	Status	Refs.
GS-0976	PubChem CID, 71528744	ACC1/2	NASH	Phase 2	[64]
			DNL	Phase 1	[65]
PF-05221304	PubChem CID, 57496611	ACC1/2	NASH	Phase 2	[66]
			Hepatic Impairment	Phase 1	[67]
MK-4074	PubChem CID, 24964679	ACC1/2	NAFLD	Phase I	[68]
^68^Ga-CBP8	^68^Ga labeled collagen binding PET imaging probe that binds to COL1A1	COL1A1	Radiation-Induced Tissue Injury	Phase 2	[94]
			Lung Cancer, and Idiopathic Pulmonary Fibrosis	Phase 1	[95]
			Myocardial Fibrosis in Cardiac Amyloidosis	Phase 3	[96]
			Early Interstitial Lung Disease	Phase 2	[97]
			The efficacy of PLN-74809 in Idiopathic Pulmonary Fibrosis	Phase 2	[98]
			The efficacy of ^64^Cu-labeled polyglucose nanoparticle (Macrin) in Cardiovascular Disease, Cancer and Sarcoidosis	Phase 1	[99]
HT-100	PubChem CID, 62894	COL1A1	DMD	Phase 1b/2a	[104]
			Solid tumors	Phase 2	[109]
			HIV-Related Kaposi's Sarcoma	Phase 2	[110]
RCT-01	Nonbulbar dermal sheath (NBDS) cells that express COL1A1 and COL2A1	COL1A1	Chronic Achilles Tendinosis	Phase 1/2	[120]
				Phase 1	[121–123]
Etomoxir	PubChem CID, 123823	CPT1A and CPT1B	Heart failure	Phase 2	[125]
Perhexiline	PubChem CID, 4746	CPT1A and CPT2	Angina pectoris	Approved	[87]
			Hypertrophic cardiomyopathy	Phase 2	[131]
Teglicar	PubChem CID, 9843897	CPT1A	T2MD	Phase 2	[140]
Recombinant human CT-1	Recombinant protein	CT-1	Reperfusion injury	Phase 2	[171]
PF-06865571	PubChem CID, 134262752	DGAT2	NAFLD, NASH	Phase 2	[75]
			Hepatic Impairment	Phase 1	[76]
			Drug‒Drug Interaction Study (Metformin)	Phase 1	[77]
IONIS-DGAT2_Rx_	Antisense oligonucleotides (ASO)	DGAT2	NAFLD	Phase 2	[78]
PF-07202954		DGAT2	NAFLD	Phase 1	[79]
PF-06427878	PubChem SID, 381129396	DGAT2	NAFLD	Phase 1	[80, 81]
CS-917					
CS-917 (the prodrug of MB05032)	PubChem CID, 9811837	FBP1	T2DM	Phase 2	[141]
MB05032					
MB07803	PubChem CID, 24770445	FBP1	T2DM	Phase 2a	[148]
Alirocumab	Monoclonal antibodies	PCSK9	Hypercholesterolemia	Approved	[164]
Evolocumab	Monoclonal antibodies	PCSK9	Hypercholesterolemia	Approved	[164]
Inclisiran	SiRNA	PCSK9	Hypercholesterolemia	Approved	[166]
AZD8233	Antisense-oligonucleotide (ASO)	PCSK9	Hypercholesterolemia	Phase 2	[167]
Civi-007	ASO	PCSK9	Hypercholesterolemia	Phase 2a	[167]
MK-0616	Polypeptide	PCSK9	Hypercholesterolemia	Phase 2	[167]
LIB003	A small recombinant fusion protein of PCSK9-binding domain (adnectin)	PCSK9	Hypercholesterolemia	Phase 3	[167, 169]
Mongersen	Antisense oligodeoxynucleotide	SMAD7	Crohn's Disease (CD)	Phase 3	[157]

The information was obtained from AdisInsight, ClinicalTrials, Glgoo, the original development company or university websites, Pubmed, and PubChem Compound

*ACC1* acetyl-CoA-carboxylase 1, *COL1A1* collagen I, *CPT1A* Carnitine palmitoyl-transferase 1A, *CT-1* cardiotrophin-1, *FBP1* fructose-1,6-bisphosphatase, *PCSK9* Proprotein convertase subtilisin/kexin type 9, *SMAD7* mothers against decapentaplegic homolog 7, *NASH* nonalcoholic steatohepatitis, *DNL* de novo lipogenesis, *NAFLD* nonalcoholic fatty liver disease, *DMD* Duchenne muscular dystrophy, *T2DM* type 2 diabetes mellitus, *CD* Crohn’s disease

**Fig. 3:**
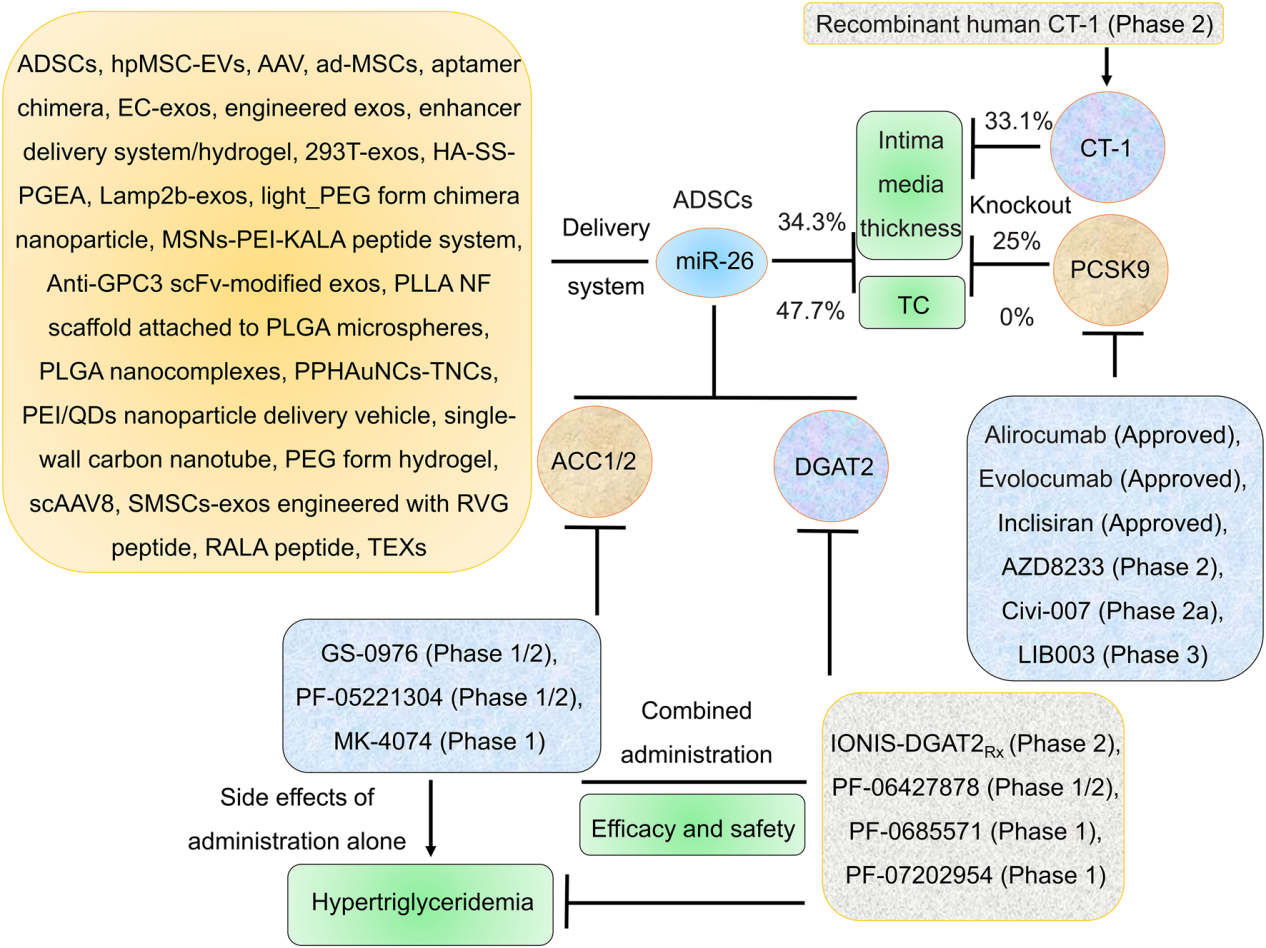
MiR-26 delivery systems and targeting ACC1/2, CT-1, DGAT2, and PCSK9 with drugs that were approved or investigated in clinical trials. ADSCs overexpressing miR-26 reduced IMT more than those with PCSK9 or CT-1 knockout. DGAT2 inhibitors can reduce the side effects of ACC1/2 inhibitors. Multiple materials have been used to deliver miR-26. *ACC1* acetyl-CoA-carboxylase 1, *CT-1* cardiotrophin-1, *PCSK9* Proprotein convertase subtilisin/kexin type 9

DGAT2 is an important enzyme for TG biosynthesis (up to 90%) [73, 74]. Many DGAT2 inhibitors, such as PF-06865571 (also named ervogastat) [75–77], IONIS-DGAT2_Rx_ (also named ISIS 484137) [78], PF-07202954 [79], PF-06427878 [80, 81], PF-06424439 [82], and benzimidazolone derivatives [83], have been investigated in preclinical and clinical trials. Importantly, DGAT2 inhibitors can reduce the side effects of ACC1/2 inhibitors. PF-06865571 combined with PF-05221304 showed good efficacy and safety in a phase II trial for the treatment of nonalcoholic steatohepatitis (NASH) [66, 75], suggesting that the development of multitarget inhibitors is the future direction for drugs targeting DGAT2 and ACC1/2 (Fig. 3). MiR-26 suppressed ACC1/2 expression and DGAT2 expression. Therefore, miR-26 is a potential candidate for development as a multitarget inhibitor of ACC1, ACC2, and DGAT2 expression.

### COL1A1

**Fig. 4:**
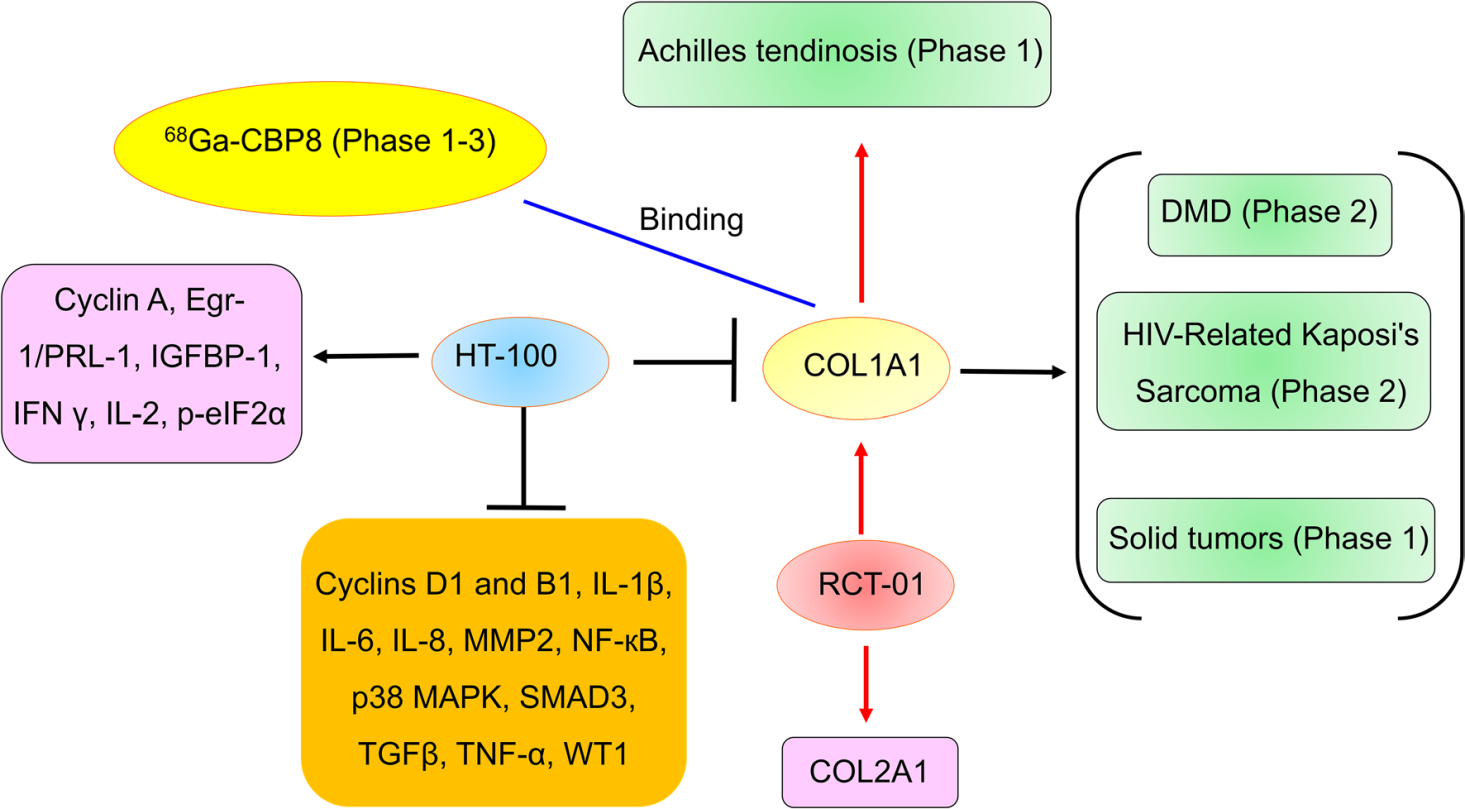
The COL1A1-specific agents entering clinical trials and their targets. *COL1A1* collagen I, *Egr-1* early growth response-1, *IFNγ* Interferon-γ, *IGFBP-1* insulin-like growth factor binding protein 1, *IL-1β* Interleukin (IL)-1 beta, *MMP2* matrix metalloproteinase 2, *NF-κB* Nuclear factor κB, *p-eIF2α* phosphorylated eIF2α, *PRL-1* phosphatase of regenerating liver-1, *SMAD3* mothers against decapentaplegic homolog 1, *TNF-α* tumor necrosis factor alpha, *WT1* Wilms’ tumor gene 1, *DMD* duchenne muscular dystrophy

COL1A1, the main fibrous collagen in the extracellular matrix (ECM), is located mainly in tendons, bones, teeth, skin, lungs, heart, and vasculature. COL1A1 regulates MMP-2, MMP-8 and MMP-9 through its bioactive peptide/fragments proline-glycine-proline (PGP), α1 C1158/59 (C-1158/59), and C-propeptide [84–86]. COL1A1 promotes tumor cell proliferation, epithelial–mesenchymal transition, and metastasis by binding to its receptor. COL1A1 also modulates the efficacy of tumor treatments, such as chemotherapy, radiation, and immunotherapy [87], suggesting that COL1A1 plays a role in tumor development. In fact, COL1A1 is key to many diseases, such as atherosclerosis, myocardial fibrosis, heart failure, and osteogenesis imperfecta [88–90]. Many COL1A1-specific agents, such as HT-100 (also named Halofuginone Hydrobromide, PCS-100, Stenorol, and Tempostatin), ^68^Ga-Collagen Binding Probe #8 (CBP8) (^68^Ga-CBP8), and RCT-01, have been investigated in clinical trials, and the results suggested that COL1A1 is a promising target for drug development (Fig. 4).

#### ^68^Ga-CBP8

^68^Ga-CBP8 is a gallium-68-labeled collagen-binding PET imaging probe that can selectively bind to COL1A1. ^68^Ga-CBP8 has a high affinity for COL1A1 in a mouse lung injury model of radiation lung injury and has good pharmacological and pharmacokinetic characteristics, with high target uptake and low retention in background tissues and organs. ^8^Ga-CBP8 has high specificity and a high target background ratio for pulmonary fibrosis in diseased animals, and it can be used to effectively detect pulmonary fibrosis and its response to therapy [91–93]. ^68^Ga-CBP8 can be used for noninvasive COL1A1 imaging in a range of human fibrotic diseases. Many clinical trials have investigated ^68^Ga-CBP8 (Table 2) [94–99]. In phase 1 clinical trials, the average injection activity of ^68^Ga-CBP8 in healthy volunteers (5 males and 4 females) was 220 Mbp. No adverse reactions were associated with the probe injection. 68Ga-CBP8 exhibited an extracellular distribution and was rapidly cleared, primarily by the kidneys. The initial distribution half-life and elimination half-life of ^68^Ga-CBP8 were 4.9 min and 72 min, respectively [100]. These results supported the further development of ^68^Ga-CBP8.

#### HT-100

HT-100, a COL1A1 inhibitor, was developed for the treatment of Duchenne muscular dystrophy (DMD) by the Akashi Therapeutics pipeline and Collgard Biopharmaceuticals [101–103]. The interim phase 1b/2a clinical data of the HT-100 trial showed improved muscle strength at low doses (N = 10). The average increase in total muscle strength was 11.7%, which was 22.3% higher than that in the steroid treatment group. No serious adverse events were associated with the drugs [104]. However, at higher doses, one patient experienced serious adverse events leading to death [105]. The further development of HT-100 for the treatment of DMD was discontinued [105–108]. HT-100 for the treatment of HIV-related Kaposi’s sarcoma (phase 2) and solid tumors (phase 1) was administered on 5 June 2013 and 24 July 2012, respectively [109, 110], but no results were disclosed. The further development of HT-100 in many diseases, including bladder cancer, cancer, coronary artery restenosis, hepatic fibrosis, kidney disorders, renal fibrosis, and scleroderma, has been discontinued [103]. Notably, further development of HT-100 in DMD patients may still be in phase 2 trials as of September 2022 [103]. Despite our best efforts, we did not find any information on the outcomes. In addition, HT-100 reduced the expression of multiple genes, including cyclins D1 and B1, IL-1β, IL-6, IL-8, MMP2, NF-κB, p38 MAPK, SMAD3, TGFβ, TNF-α, and Wilms’ tumor gene 1 (WT1), and increased the expression of multiple genes, including cyclin A, early growth response-1 (Egr-1)/phosphatase of regenerating liver-1 (PRL-1), IFNγ, IL-2, and phosphorylated eIF2α (p-eIF2α) [111–117]. Therefore, we should be wary of the low specificity and off-target effects of HT-100, which may result in high toxicity.

#### RCT-01

RCT-01, an autologous cell therapy, consists nonbulbar dermal sheath (NBDS) cells that expressed COL1A1 and collagen II (COL2A1). NBDS cells are fibroblasts isolated from a small tissue sample at the back of a patient's scalp (hair follicles). NBDS cells express the highest levels of COL1A1 and COL2A1 among fibroblasts anywhere in the body. These cells can repair and regenerate tissues. RCT-01 was developed for the treatment of Achilles tendinosis by RepliCel and YOFOTO in Greater China and by RepliCel elsewhere in the world. A phase 1 clinical study of RCT-01 demonstrated its safety. RCT-01 regenerated tendon tissue, restored blood flow, reduced pain, and improved function in patients with chronic tendinopathy [118, 119]. However, RCT-01 did not have significantly better efficacy than standard therapy in phase 1 studies [119]. The study was also discontinued due to slow enrollment on 28 September 2017 [120]. Notably, in 2021, RepliCel announced that it would begin a second clinical study of RCT-01 in Japan [121]. RCT-01 was reviewed by the Pharmaceutical and Medical Devices Agency (PMDA) of Japan on 28 July 2022 [122] and is expected to receive clinical registration soon. This clinical protocol for the treatment of chronic tendinopathies (including Achilles tendinopathy) was nearing completion as of 28 November 2022 [123].

### CPT1A

**Fig. 5:**
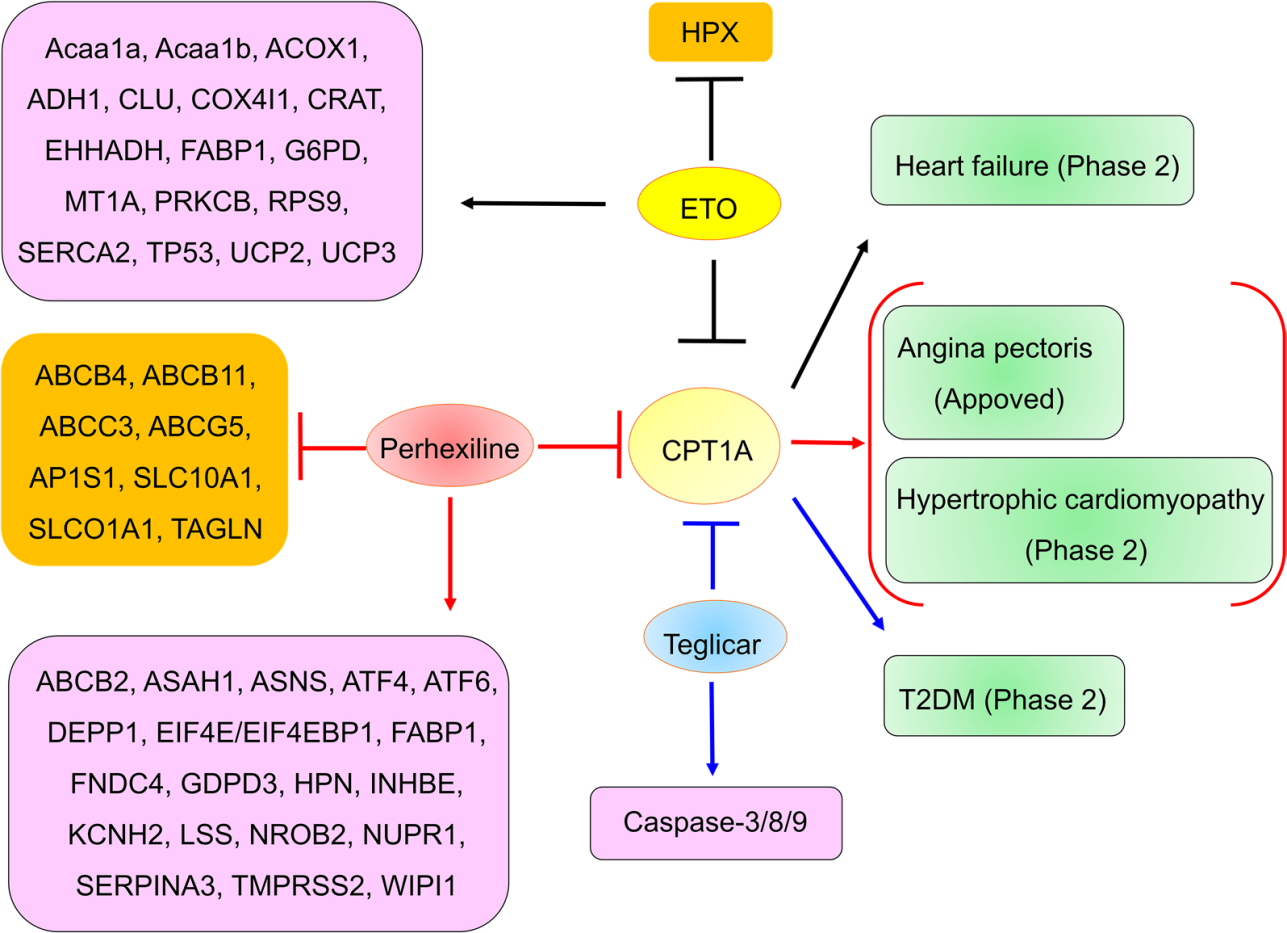
The CPT1A inhibitors entering clinical trials and their targets. *Acaa1a* acetyl-CoA acyltransferase, *ACOX1* acyl-CoA oxidase 1, *ADH1* alcohol dehydrogenase 1, *AP1S1* adaptor related protein complex 1 subunit sigma 1, *ASAH1* *N*-acylsphingosine amidohydrolase 1, *ASNS* asparagine synthetase, *ATF4* activating transcription factor 4, *CLU* clusterin, *COX4I1* matrix cytochrome C oxidase subunit 4I1, *CRAT* carnitine acetyltransferase, *DEPP1* DEPP autophagy regulator 1, *EHHADH* enoyl-CoA hydratase and 3-hydroxyacyl CoA dehydrogenase, *EIF4EBP1* eukaryotic translation initiation factor 4E binding protein 1, *FABP1* fatty acid binding protein 1, *FNDC4* fibronectin type III domain containing 4, *GDPD3* glycerophosphodiester phosphodiesterase domain containing 3, *G6PD* glucose-6-phosphate dehydrogenase, *HPN* hepsin, *HPX* hemopexin, *INHBE* inhibin subunit beta E, *KCNH2* potassium voltage-gated channel subfamily H member 2, *LSS* lanosterol synthase, *MT1A* metallothionein 1A, *NROB2* nuclear receptor subfamily 0 group B member 2, *NUPR1* nuclear protein 1, transcriptional regulator, *PRKCB* protein kinase C beta, *RPS9* ribosomal protein S9, *SERCA2* sarco-endoplasmic reticulum calcium ATPase 2, *SERPINA3* serpin family A member 3, *SLC10A1* solute carrier family 10 member 1, *TAGLN* transgelin, *TMPRSS2* transmembrane serine protease 2, *TP53* tumor protein p53, *UCP2* uncoupling protein 2, *WIPI1* WD repeat domain, phosphoinositide interacting 1, *T2DM* type 2 diabetes mellitus

CPT1A is the rate-limiting enzyme of fatty acid β-oxidation and long-chain fatty acid (LC-FAO) oxidation. CPT1A-mediated LC-FAO is essential for the development of CD8+ T-cell memory and protective immunity, which play key roles in the adaptive immune response against infection and cancer [124, 125]. Many CPT1A inhibitors, such as etomoxir (ETO), perhexiline (also named Pexsig), and teglicar (also named ST1326), have been investigated in clinical trials, suggesting that CPT1A is a promising target for drug development (Fig. 5).

#### ETO

ETO inhibits CPT1A expression by binding to its active site. ETO also inhibits the expression of CPT1B, suggesting that ETO has weak specificity for CPT1A [125]. The use of ETO for the treatment of heart failure has been investigated in phase 2 clinical trials, but further development of ETO in heart failure and T2DM patients was discontinued due to hepatotoxicity [125, 126]. ETO can increase the expression of multiple genes, including acetyl-CoA acyltransferase (Acaa1a), Acaa1b, acyl-CoA oxidase 1 (ACOX1), alcohol dehydrogenase 1 (ADH1), clusterin (CLU), matrix cytochrome C oxidase subunit 4I1 (COX4I1), carnitine acetyltransferase (CRAT), enoyl-CoA hydratase and 3-hydroxyacyl CoA dehydrogenase (EHHADH), fatty acid binding protein 1 (FABP1), glucose-6-phosphate dehydrogenase (G6PD), metallothionein 1A (MT1A), protein kinase C beta (PRKCB), ribosomal protein S9 (RPS9), sarco-endoplasmic reticulum calcium ATPase 2 (SERCA2, encoding ATP2A2), tumor protein p53 (TP53), uncoupling protein 2 (UCP2), and UCP3, and decrease hemopexin (HPX) expression [127–130]. Therefore, the low specificity of perhexiline and its off-target effects may have led to its high toxicity and cessation of development.

#### Perhexiline

Perhexiline is a CPT1A and CPT2 inhibitor but has less of an effect on CPT2. Perhexiline was approved for the treatment of angina pectoris. However, long-term use of perhexiline can cause neurotoxicity and hepatotoxicity [87]. The use of perhexiline for the treatment of hypertrophic cardiomyopathy was also investigated in clinical trials. Its further study in a phase 2 clinical trial was terminated due to a lack of efficacy on 31 August 2017 [131]. In fact, perhexiline decreased multiple genes expression, including ABCB4, ABCB11, ABCC3, ABCG5, adaptor related protein complex 1 subunit sigma 1 (AP1S1), solute carrier family 10 member 1 (SLC10A1), solute carrier organic anion transporter family, member 1a1 (SLCO1A1), and transgelin (TAGLN), and increased multiple genes expression, including ABCB2, *N*-acylsphingosine amidohydrolase 1 (ASAH1), asparagine synthetase (ASNS), activating transcription factor 4 (ATF4), ATF6, DEPP autophagy regulator 1 (DEPP1), eukaryotic translation initiation factor 4E (EIF4E)/EIF4E binding protein 1 (EIF4EBP1), FABP1, fibronectin type III domain containing 4 (FNDC4), glycerophosphodiester phosphodiesterase domain containing 3 (GDPD3), hepsin (HPN), inhibin subunit beta E (INHBE), potassium voltage-gated channel subfamily H member 2 (KCNH2), lanosterol synthase (LSS), nuclear receptor subfamily 0 group B member 2 (NROB2), nuclear protein 1, transcriptional regulator (NUPR1), serpin family A member 3 (SERPINA3), transmembrane serine protease 2 (TMPRSS2), WD repeat domain, phosphoinositide interacting 1 (WIPI1) [132–137]. The low specificity of perhexiline and its off-target effects may have led to its high toxicity.

#### Teglicar

Teglicar, an aminocarnitine analog, is a formylcarnitine derivative. Teglicar was more selective for CPT1A than for ETO. Teglicar significantly improved hyperglycemia and regulated glucose homeostasis in obesity and type 2 diabetes mouse models [138]. Teglicar also inhibited the growth of canine breast cancer cells by inducing caspase-3/8/9-mediated apoptosis [87]. Teglicar for the treatment of T2DM (40 males and females) was well tolerated and safe in phase 1 clinical trials. The half-life of teglicar was 25 h. Teglicar (450 mg/day) significantly improved insulin resistance (from 4.1 on day 1 to 3.0 on day 16) and blood sugar levels (reducing 16 mg/dl on day 16 vs. 4 mg/dl placebo group) [139]. These results supported the further development of Teglicar, but its further development for the treatment of T2DM in phase 2 clinical trials was discontinued [140].

### FBP1

**Fig. 6:**
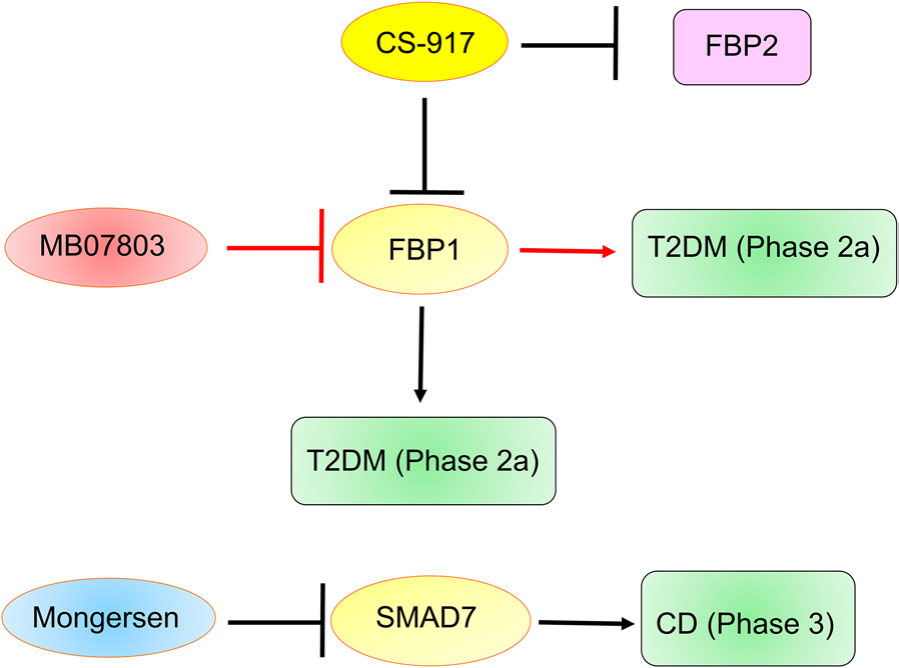
The FBP1 inhibitors and SMAD7 inhibitors entering clinical trials. *FBP1* fructose-1,6-bisphosphatase, *SMAD7* mothers against decapentaplegic homolog 7, *T2DM* type 2 diabetes mellitus, *CD* Crohn’s disease

FBP1 is a rate-controlling enzyme of gluconeogenesis [141]. Many FBP1 inhibitors, such as CS-917 (also named MB06322) and MB07803 (also named VK0612), were investigated in clinical trials, suggesting that FBP1 is a promising target for drug development (Fig. 6).

#### CS-917

CS-917 is a prodrug of MB05032 (also named R-125338). MB05032 inhibits FBP1 expression by binding to its active AMP site [142]. MB05032 has low cell penetration and oral absorption due to its phosphate group. Thanks to its dialanyl amide group, CS-917 administration leads to much higher bioavailability of MB05032 than direct administration of MB05032. CS-917 is catalyzed to an intermediate form, R-134450, by the esterase enzyme and then to an active form, MB05032, by the phosphoramidase enzyme [143]. CS-917 has inhibited gluconeogenesis and improved fasting and postprandial hyperglycemia in preclinical trials [142, 144, 145]. Six phase 1/2 clinical trials of MB07803 have been completed [146]. The main theoretical side effect of FBP1 inhibitors is an increased risk of hypoglycemia. However, CS-917 exhibited good tolerability and safety in overnight-fasted healthy volunteers (in phase 1 clinical trials) and patients with T2DM (in phase 2 clinical trials). CS-917 at doses of 50, 200, and 400 mg/d reduced glucose levels in patients with T2DM. However, at a dose of 100 mg/d, CS-917 did not affect glucose, suggesting that the efficacy of CS-917 was not dose dependent. CS-917 at doses of 50 and 100 mg/d also did not affect glucose levels in phase 2a clinical trials. The lactic acidosis caused by CS-917 in combination with metformin was significantly resolved after withdrawal in phase 1 clinical trials, suggesting a possible interaction between metformin and CS-917. Further development of CS-917 has been halted due to concerns about its efficacy and interactions [141]. CS-917 also decreased the FBP2 level [147], suggesting that the low specificity of CS-917 and its off-target effects may have led to the suspension of its development.

#### MB07803

MB07803, a derivative of CS-917, was developed by Viking Therapeutics. MB07803 has better pharmacokinetic properties than CS-917, including higher oral bioavailability, longer active metabolite half-life, and lower inactive *N*-acetylated metabolite production. Six phase 1 clinical trials and one phase 2a clinical trial on MB07803 have been done [148]. MB07803 exhibited good safety and tolerability in healthy volunteers (in phase 1 clinical trials) and patients with T2DM (in phase 2a clinical trials). MB07803 reduced fasting blood sugar (FBG) at the highest dose (200 mg/day). P.O. 28 days). After optimal dosing, MB07803 also exhibited good tolerability and safety. The maximum tolerated dose of MB07803 in phase 1b was 200 mg twice daily. MB07803 reduced FBG in a dose-dependent manner (50, 200, and 400 mg BID) [149]. These results supported the further development of MB07803. Even so, no FBP1 inhibitors have been approved. More studies are needed to confirm the efficacy of MB07803 and the risks associated with its combination with other drugs, such as metformin.

### SMAD7

SMAD7, a member of the I-Smad family, enhances proinflammatory cytokine expression by inhibiting the NF-κB and TGF-β1 signaling pathways and controlling DNA promoter activity [150, 151]. SMAD7 is involved in many diseases, such as cardiovascular diseases, autoimmune diseases, inflammatory diseases, cancers, and kidney diseases [152, 153]. Mongersen (also named GED-0301), an anti-SMAD7 oligonucleotide, decreased the production and activity of SMAD7 by promoting RNase H-mediated degradation of SMAD7 mRNA (Fig. 6). Mongersen was investigated for the treatment of Crohn’s disease (CD) and ulcerative colitis (UC) in clinical trials [154]. Mongersen exhibited good efficacy and safety for the treatment of CD in one phase 1 and three phase 2 clinical trials [155–158], but its development was suspended due to a lack of efficacy in phase 3 clinical trials. Notably, the chemical and pharmacological properties of mongersen are extremely unstable. Phase 1 clinical trials and phase 2 clinical trials used the same batch of mongersen, while phase 3 clinical trials used batches that were mostly different from phases 1 and 2. Most mongersen from phase 3 clinical trials did not inhibit SMAD7 expression in vitro [152, 159, 160]. In particular, the results of one phase 2 clinical trial were released after the phase 3 clinical trial. Mongersen showed good efficacy and safety in this trial. Mongersen decreased the percentage of CCR9−expressing CD45+ cells [157]. CCR9, a chemokine receptor induced by TGFβ1, induces white blood cells in lymphoid tissue to home to the intestinal mucosa, promoting the development of inflammation. CCR9-positive cells are markers of CD [161]. Mongersen inhibited SMAD7 expression because the batch used was also different from that used in the phase 3 clinical trial. This solved the stability problem of mongersen. Its development may be able to continue.

## The potential value of miR-26 in the development of drugs to treat atherosclerosis

The administration of ADSCs overexpressing miR-26 (IV, 1 × 10^10^/mouse) for two weeks in a CAS model (apoE−/− mice fed a rich-fat diet (21% fat and 0.2% cholesterol) for 12 weeks) reduced carotid intima–media thickness (IMT), TC, TG, and LDL-C by 34.3%, 47.7%, 35.7%, and 22.9%, respectively [36]. Proprotein convertase subtilisin/kexin type 9 (PCSK9) knockout in apoE−/− mice fed a regular diet (6% fat and 0% cholesterol) for 6 months did not change IMT or TC levels. PCSK9 knockout in apoE−/− mice fed a Western diet (21% fat and 0.2% cholesterol) for 6 months reduced the IMT of valves, the root, the ascending aorta, and the brachiocephalic artery (BCA) by 25%, 18%, 9%, and 0%, respectively, but did not change TC levels [162]. Knockout of cardiotrophin-1 (CT-1), an IL-6 family member, reduced the media thickness of the carotid and aortic arteries by 33.1% and 20.5%, respectively, in mice [163]. These results suggest that the efficacy of ADSCs overexpressing miR-26 in reducing IMT is higher than that of those with PCSK9 or CT-1 knockout. Interestingly, many drugs targeting PCSK9, including alirocumab, evolocumab, inclisiran (also named ALN-60212), AZD8233, Civi-007, MK-0616, and LIB003 (also named lerodalcibep), have been approved or investigated in clinical trials [164–170]. The targeting of CT-1 with recombinant human CT-1 was investigated in clinical trials [171], and the results suggested that, like CT-1 and PCSK9, miR-26 has potential for drug development (Fig. 3). MiR-26 has not been studied in depth in atherosclerosis in vivo, so more studies are needed.

## Materials for delivering miR-26

Multiple materials have been used to deliver miR-26 (Table 3), including ADSCs, hpMSC-EVs, adeno-associated virus (AAV) [172, 173], aptamer chimeras [174], EC-derived exos (EC-exos) [175], engineered exos [176], enhancer delivery systems/hydrogels [177], exos derived from 293T cells [178], HA-SS-PGEA [179], Lamp2b-exos [180], light polyethylene glycol (PEG) chimera nanoparticles [181], a mesoporous silicon nanoparticle (MSN)-polyethylenimine (PEI)-KALA peptide system [182], anti-Glypican 3 (GPC3) single-stranded variable fragment (scFv)-modified exos [183], a poly(l-lactic acid) (PLLA) nanofibrous (NF) scaffold attached to poly(lactic-co-glycolic acid) (PLGA) microspheres [184], PLGA nanocomplexes [185], polyetherimide-conjugated PEGylated gold nanocage ternary nanocomplexes (PPHAuNCs-TNCs) [186], a PEI/Quantum dot (QD) nanoparticle delivery vehicle [187], single-wall carbon nanotubes (SWCNTs) [188], PEG hydrogels [189], self-complementary AAV serotype 8 (scAAV8) [190], skeletal muscle satellite cell-derived exos (SMSCs-exos) engineered with RVG peptide [191], RALA peptide [192], and tumor cell-derived exos (TEXs) [193]. More studies are needed to confirm which materials are more suitable for mass production and clinical application.

**Table 3:** MiR-26 delivery systems and concentrations

Deliver materials	Concentration	Refs.
ADSCs	Transfected with miR-26-promoter	[36]
HpMSC-EVs	Unknown	WO2021225214
Self-complementary AAV (scAAV)	Unknown	[172]
AAV	40 nM	[173]
Aptamer chimera	33.5 pmol/g	[174]
EC-exos	100 nM	[175]
Engineered exos	0.12 pmol/μg	[176]
Enhancer delivery system, including HyStem-HP hydrogel, hBMMSCs, and Cy-3-labeled agomir (miRNA enhancer)	50 nM	[177]
293 T-exos	100 μg	[178]
PEI, PGEA, and HA-SS-PGEA	60 pmol	[179]
Lamp2b-exos	40 µg (exosomes)	[180]
Light_PEG chimera nanoparticles	0.9 mg/kg (Pharmacokinetics, BALB/c mice), 2.4 mg/kg (breast cancer models)	[181]
MSNs-PEI-KALA peptide system	0.1 nmol/μL	[182]
Anti-GPC3 scFv-modified exos	20 nM	[183]
PLLA NF scaffold attached to PLGA microspheres	60 pmol	[184]
PLGA nanocomplexes	20 ng/μL	[185]
PPHAuNCs-TNCs	2.2 mg/kg	[186]
PEI/QDs nanoparticle delivery vehicle	1 µg	[187]
SWCNT	40 mg/mL (SWCNT-miR-26a)	[188]
PEG hydrogels	0.06 pmol/μL	[189]
ScAAV8	Unknown	[190]
SMSCs-exos engineered with RVG peptide	100 µg/week (RVG-miR-26a-Exos)	[191]
RALA peptide	50 µg/mL	[192]
TEXs	1 nmol/μg exosomes	[193]

## Patents related to miR-26

Targeting miR-26 (a mimic) for the treatment of bone injury was investigated in preclinical trials by Air Force Medical University in China (patent number: CN104694542A). The delivery of miR-26 via human placental mesenchymal stem cell (hpMSC)-derived extracellular vesicles (EVs) for the treatment of coronavirus infection and autoimmune disease was investigated in preclinical trials by CHA University and Ts Cell Bio Co., Ltd. (patent number: WO2021225214). Targeting miR-26 (an inhibitor) for the treatment of ocular disease was investigated in preclinical trials by the University of Massachusetts (patent number: WO2021178668). MiR-26 has many target genes, and avoiding the occurrence of off-target effects requires further study. In addition, the diagnostic value of CTDSPL (host gene of miR-26a-1) in patients with diabetes (patent number: WO2015084862A1) or the chemotherapeutic resistance of triple-negative breast cancer (TNBC) (patent number: WO2023099889A1) was investigated at Wayne State University and Queens University of Belfast. Targeted inhibition of CTDSP1 (host gene of miR-26b) for the treatment of glioblastoma [194], brain tumors (patent number: CN113004356A), and neurodegenerative diseases (patent number: CN113004356A) was investigated at Purdue University, the University of California San Diego, the University of Texas at Austin, and Shanghai Jiao Tong University. However, the effect of miR-26 on host genes was not investigated.

## Conclusions and future directions

MiR-26 decreases atherosclerosis development in vivo by suppressing the expression of multiple genes, including ACC1, ACC2, ACLY, ACSL3, ACSL4, ALDH3A2, ALPL, BMP2, CD36, COL1A1, CPT1A, CTGF, DGAT2, EHHADH, FAS, FBP1, GATA4, GSK3β, G6PC, Gys2, HMGA1, HMGB1, LDLR, LIPC, IL-1β, IL-6, JAG2, KCNJ2, MALT1, β-MHC, NF-κB, PCK1, PLCβ1, PYGL, RUNX2, SCD1, SMAD1, SMAD4, SMAD5, SMAD7, SREBF1, TAB3, TAK1, TCF7L2, and TNF-α. Several interesting and critical tasks remain to be explored: (1) miR-26 also promotes foam cell formation by reducing ABCA1 and ARL4C expression in vitro. More studies are needed to confirm the role of miR-26 in foam cell formation in vivo. (2) The miR-26 level in ADSC-exos is a potential biomarker for the diagnosis of lipid metabolism disorders and inflammatory states. However, biomarkers should be chosen considering disease status, the predisease state, or prognosis and should also be sensitive, specific, and superior to existing markers. (3) IFN-α therapy is effective in patients with HCC with low expression of miR-26. Therefore, miR-26 is expected to be a marker of IFN-α therapy for the treatment of HCC. In the future, we should determine the range of miR-26 expression levels in HCC to confirm when IFN-α therapy should be used. (4) IFN-α therapy has been approved for the treatment of multiple diseases, such as viral infections, hematological diseases, and various cancers. However, the role of miR-26 in IFN-α therapy for the treatment of these diseases is unclear. (5) The delivery of miR-26 to specific tissue- or cell-specific niches has been investigated by using different materials. It is still unclear which materials are most suitable for mass production and clinical applications. (6) MiR-26 target genes, such as ACC1, ACC2, COL1A1, CPT1A, DGAT2, FBP1, and SMAD7, are promising targets for drug development. Hypertriglyceridemia is a common adverse effect of ACC1/2 inhibitors that limits their clinical development. DGAT2 inhibitors can reduce the side effects of ACC1/2 inhibitors. Therefore, miR-26 is a potential candidate for development as a multitarget inhibitor of ACC1, ACC2, and DGAT2 expression. Like PCSK9 and CT-1, miR-26 is a promising candidate for drug development, but evidence on it is still limited. (7) COL1A1-specific agents, such as HT-100, RCT-01, and ^68^Ga-CBP8, are still being tested in clinical trials. No COL1A1-specific agents have been approved for use. (8) Perhexiline, a CPT1A and CPT2 inhibitor, has been approved, but the development of CPT1A inhibitors has not been successful. For example, the CPT1A inhibitor ETO was discontinued due to safety concerns, and teglicar was discontinued for unknown reasons. Investigating the underlying mechanism is conducive to the further development of teglicar. (9) The FBP1 inhibitor MB07803 exhibited good efficacy in phase 1b clinical trials, supporting its further development. Further development of its derivative CS-917 was discontinued due to efficacy and drug interactions. It is not known whether MB07803 causes drug interactions. (10) The SMAD7 inhibitor mongersen was discontinued due to poor efficacy. The quality of mongersen drug batches is not uniform, and some even have no inhibitory effect on SMAD7. Improving the quality of mongersen is the key to its development. (11) No miR-26 agents have been investigated in clinical trials. In addition, miR-26 has many target genes. We should consider which of these are the main target genes, where they are expressed, whether low doses of miR-26 cause changes in their expression, and how to deliver miR-26.

In summary, the detection of miR-26 in IFN-α therapy is worthy of further development. Targeting ACC1, ACC2, COL1A1, CPT1A, DGAT2, FBP1, and SMAD7 could improve the success rate of drug development. As research continues and technology advances, we believe that new drugs will be developed to combat atherosclerotic CVD. We have done our best not to overlook important contributions and to present the cited results as accurately as possible. If have, we apologize for the omission or error.

## Data Availability

Not applicable.

## Abbreviations

CVD: Cardiovascular disease
MI: Myocardial infarction
IHD: Ischemic heart disease
LDL: Low-density lipoprotein
ROS: Reactive oxygen species
VSMCs: Vascular smooth muscle cells
MMP2: Metalloproteinase 2
CTDSPL: C-terminal domain RNA polymerase II polypeptide A small phosphatase-like
CTDSP2: C-terminal domain RNA polymerase II polypeptide A small phosphatase 2
ABCA1: ATP-binding cassette transporter A1
ACC1: Acetyl-CoA-carboxylase 1
ACLY: ATP-citrate lyase
ACSL3: Acyl-CoA synthetase long-chain 3
ALDH3A2: Aldehyde dehydrogenase 3 family member A2
FALDH: Fatty aldehyde dehydrogenase
ALPL: Alkaline phosphatase
ARL7: ARF-like 7
ARL4C: ADP-ribosylation factor-like 4C
β-MHC: β-Myosin heavy chain
BMP2: Bone morphogenetic protein 2
CD36: Cluster of differentiation 36
COL1A1: Collagen I
CPT1A: Carnitine palmitoyl-transferase 1A
CTGF: Connective tissue growth factor
DGAT2: Diacylglycerol acyltransferase 2
EHHADH: Enoyl-CoA hydratase/3-hydroxyacyl-CoA dehydrogenase
FASN: Fatty acid synthase
FBP1: Fructose-1,6-bisphosphatase
GATA4: GATA binding protein 4
G6PC: Glucose-6-phosphatase
GSK-3β: Glycogen synthase kinase-3β
Gys2: Glycogen synthase 2
HMGA1: High mobility group A1
IL-1β: Interleukin (IL)-1 beta
JAG2: Jagged canonical Notch ligand 2
KCNJ_2_: Potassium inwardly rectifying channel subfamily J member 2
LDLR: Low-density lipoprotein receptor
LIPC: Lipase C hepatic type
MALT1: Mucosa-associated lymphoid tissue lymphoma translocation protein 1
NF-κB: Nuclear factor κB
PCK1: Carboxykinase
PLCβ1: Phospholipase C beta
PYGL: Glycogen phosphorylase L
RUNX2: Runt-related protein 2
SCD1: Stearoyl-coenzyme A desaturase 1
SMAD1: Mothers against decapentaplegic homolog 1
SREBF1: Sterol regulatory element-binding transcription factor 1
TAB3: TGFβ-activated kinase binding protein 3
TAK1: TGFβ-activated kinase-1
TCF7L2: Transcription factor 7 like 2
TNF-α: Tumor necrosis factor alpha
TC: Total cholesterol
CAS: Carotid atherosclerosis
ADSCs-exos: Adipose-derived stem cells
HL: Hepatic lipase
ANGPTL3: Angiopoietin-like 3
IFN-α: Interferon-α
HCC: Hepatocellular carcinoma
RCC: Renal cell cancer
EZH2: Zeste homolog 2
USP3: Ubiquitin-specific protease 3
PNPT1: Polyribonucleotide nucleotidyltransferase 1
MINOCA: Nonobstructive coronary arteries
EHOA: Erosive hand osteoarthritis
PsA: Psoriatic arthritis
EC: Endothelial cell
AF: Atrial fibrillation
FA: Fatty acid
IMT: Carotid intima-media thickness
PCSK9: Proprotein convertase subtilisin/kexin type 9
BCA: Brachiocephalic artery
CT-1: Cardiotrophin-1
AAV: Adeno-associated virus
EC-exos: EC-derived exos
PEG: Light_polyethylene glycol
MSN: Mesoporous silicon nanoparticle
PEI: Polyethylenimine
GPC3: Glypican 3
scFv: Single-stranded variable fragment
PLLA: Poly(l-lactic acid)
NF: Nanofibrous
PLGA: Poly(lactic-co-glycolic acid)
PPHAuNCs-TNCs: PEGylated gold nanocage ternary nanocomplexes
QD: Quantum dot
SWCNTs: Single-wall carbon nanotubes
scAAV8: Self-complementary AAV serotype 8
SMSCs-exos: Skeletal muscle satellite cell-derived exos
TEXs: Tumor cell-derived exos
hpMSC: Human placental mesenchymal stem cell
EVs: Extracellular vesicles
TNBC: Triple-negative breast cancer
NASH: Nonalcoholic steatohepatitis
ECM: Extracellular matrix
PGP: Peptide/fragments proline–glycine–proline
^68^Ga-CBP8: ^68^Ga-Collagen Binding Probe #8
DMD: Duchenne muscular dystrophy
WT1: Wilms’ tumor gene 1
Egr-1: Early growth response-1
PRL-1: Phosphatase of regenerating liver-1
IGFBP-1: Insulin-like growth factor binding protein 1
p-eIF2α: Phosphorylated eIF2α
NBDS: Nonbulbar dermal sheath
COL2A1: Collagen II
PMDA: Pharmaceutical and Medical Devices Agency
LC-FAO: Long-chain fatty acid
ETO: Etomoxir
T2DM: Type 2 diabetes mellitus
Acaa1a: Acetyl-CoA acyltransferase
ACOX1: Acyl-CoA oxidase 1
ADH1: Alcohol dehydrogenase 1
CLU: Clusterin
COX4I1: Matrix cytochrome C oxidase subunit 4I1
CRAT: Carnitine acetyltransferase
EHHADH: Enoyl-CoA hydratase and 3-hydroxyacyl CoA dehydrogenase
FABP1: Fatty acid binding protein 1
G6PD: Glucose-6-phosphate dehydrogenase
MT1A: Metallothionein 1A
PRKCB: Protein kinase C beta
RPS9: Ribosomal protein S9
SERCA2: Sarco-endoplasmic reticulum calcium ATPase 2
TP53: Tumor protein p53
UCP2: Uncoupling protein 2
HPX: Hemopexin
AP1S1: Adaptor related protein complex 1 subunit sigma 1
SLC10A1: Solute carrier family 10 member 1
SLCO1A1: Solute carrier organic anion transporter family, member 1a1
TAGLN: Transgelin
ASAH1: *N*-Acylsphingosine amidohydrolase 1
ASNS: Asparagine synthetase
ATF4: Activating transcription factor 4
DEPP1: DEPP autophagy regulator 1
EIF4E: Eukaryotic translation initiation factor 4E
EIF4EBP1: EIF4E binding protein 1
FNDC4: Fibronectin type III domain containing 4
GDPD3: Glycerophosphodiester phosphodiesterase domain containing 3
HPN: Hepsin
INHBE: Inhibin subunit beta E
KCNH2: Potassium voltage-gated channel subfamily H member 2
LSS: Lanosterol synthase
NROB2: Nuclear receptor subfamily 0 group B member 2
NUPR1: Nuclear protein 1, transcriptional regulator
SERPINA3: Serpin family A member 3
TMPRSS2: Transmembrane serine protease 2
WIPI1: WD repeat domain, phosphoinositide interacting 1
FBG: Fasting blood sugar
CD: Crohn’s disease
UC: Ulcerative colitis
